# The protection of CoronaVac against the infection of wild‐type SARS‐CoV‐2 (WH‐09) or Omicron variant in nude‐hACE2 mice

**DOI:** 10.1002/ame2.12336

**Published:** 2023-07-10

**Authors:** Kaili Lin, Meixuan Liu, Lu Sun, Hanjun Fu, Hongwei Qiao, Shunyi Wang, Sidan Pan, Hong Gao

**Affiliations:** ^1^ Institute of Laboratory Animal Sciences Chinese Academy of Medical Sciences and Peking Union Medical College Beijing China

**Keywords:** immunocompromised, nude‐hACE2 mice, protection, severe acute respiratory syndrome coronavirus 2

## Abstract

**Background:**

Immunocompromised individuals have an increased risk of severe acute respiratory syndrome coronavirus 2 (SARS‐CoV‐2) infection and severe outcomes, but we pay less attention to these people. Athymic nude mice are a murine strain with a spontaneous deficiency of the *Foxn1* gene, which can result in thymic degeneration or its absence, leading to immunosuppression and a decrease in the number of T cells, and are widely used in preclinical evaluations of disease in immunocompromised populations.

**Methods:**

We investigated the protection of the coronavirus disease 2019 (COVID‐19) inactivated vaccine (CoronaVac) against the infection of wild‐type SARS‐CoV‐2 (WH‐09) or Omicron variant utilizing a hybrid‐type nude‐hACE2 mouse model.

**Results:**

Compared with nude‐hACE2/W mice, the viral load in the brain and lung tissue of nude‐hACE2 mice (nude‐hACE2/WV) infected with WH‐09 after vaccination significantly decreased, and the histopathological changes were also reduced. The viral load in the brain and lung tissue of nude‐hACE2 mice (nude‐hACE2/OV) infected with the Omicron variant after vaccination was lower than that in nude‐hACE2/O, but histopathological symptoms did not improve significantly.

**Conclusion:**

CoronaVac provides some protection against infection of both WH‐09 and the Omicron variant in the nude‐hACE2 mice. Our findings aimed to provide a reference for vaccination against SARS‐CoV‐2 in immunocompromised populations.

## Graphical abstract

We investigated the protective capability of CoronaVac against the infection of wild‐type SARS‐CoV‐2 (WH‐09) or Omicron variant utilizing a hybrid‐type nude‐hACE2 mice model. Three‐4‐month‐old nude‐hACE2 mice were immunized two or three times with 0.1 mL CoronaVac 100 TCID_50_ of WH‐09 or Omicron variant were intranasally inoculated under anesthesia by tribromoethanol (200 mg/kg) after the last dose of vaccination. Bodyweight and disease signs of the six infected animals randomly selected in each group was monitored for 5 days upon virus inoculation. Tissue samples were harvested for virological and histopathology analyses when another six mice were killed to be dying post‐infection (or 4 dpi).

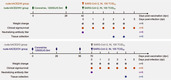

## 1 INTRODUCTION

The coronavirus disease 2019 (COVID‐19) pandemic, caused by severe acute respiratory syndrome coronavirus 2 (SARS‐CoV‐2), began in December 2019. As of June 2023, there have been over 768 million confirmed cases and 6.95 million deaths.^1^ This pandemic has put enormous pressure on and resulted in disastrous consequences to the global public health and medical systems. SARS‐CoV‐2 is a new human pathogenic *β* coronavirus that emerged from animal hosts.^2^ Patients infected with SARS‐CoV‐2 may experience fever and respiratory symptoms, with most patients recovering within 1–2 weeks. However, some patients may progress to acute respiratory distress syndrome, multiple organ failure, and death.^3^ The mass inoculation of a COVID‐19 vaccine, inducing herd immunity, is considered a committed step toward ending the current global pandemic. However, several special populations, including those with weakened immunity and immune deficiency, were not included in sufficient numbers in the clinical trials of COVID‐19 vaccines^.^ ^4^, ^5^, ^6^ One of the important reasons for excluding immune‐deficient individuals is that immune deficiency can impair the humoral immune responses and cellular immune responses induced by vaccines, making it difficult to measure the effectiveness on the immune system.^7^ The World Health Organization states that immunization plans should give preference to vulnerable groups, including the elderly, people with immune deficiencies, and those with underlying health.^8^ It should be noted that there are many types of such diseases, and we have not yet fully understood the pathogenesis.

Athymic nude mice are a murine strain with a spontaneous deficiency of the *Foxn1* gene, which can result in thymic degeneration or its absence, leading to immunosuppression and a decrease in the number of T cells.^9^ The inherent characteristics of low immunity in nude mice was widely used to study animal immunodeficiency diseases and the abnormal response of pathogenic factors to the body. Considering that nude mice cannot be infected with WH‐09, we investigated the protective effect of CoronaVac against WH‐09 or Omicron variant infection utilizing a hybrid‐type nude‐hACE2 mouse model. Our research aimed to provide basic research datas for vaccination against SARS‐CoV‐2 among immunodeficient individuals.

## 2 METHODS

### 2.1 Viruses, vaccines, and biosafety

The SARS‐CoV‐2/human/CHN/WH‐09/2020 (GenBank: MT093631) and SARS‐CoV‐2/human/CHN/Omicron‐1/2021 (BA.1, GenBank: OM095411) used in this study were provided by the Institute of Laboratory Animal Sciences, Peking Union Medical College (ILAS and PUMC). The virus was cultured in Vero E6 cells, and the virus titers were determined using 50% tissue culture infection dose (TCID_50_). CoronaVac was produced by Sinovac Life Sciences with a labeled amount of 1200 SU/0.5 mL and a specification of 0.5 mL per dose. All experiments involving live SARS‐CoV‐2 were conducted in accordance with approved standard operating procedures in the Animal Biosafety Level 3 laboratory of ILAS and PUMC (CNAS BL0010).

### 2.2 Animals

Nude‐hACE2 (K18) mice were produced by ILAS and PUMC. F1 hybrid mice were obtained by hybridizing female hACE2^+/−^ (K18) mice with male BALB/c‐nude mice in the ratio of 1:1. F1 female hACE2^+/−^ mice identified using PCR (polymerase chain reaction) amplification of DNA and agarose gel electrophoresis were backcrossed with male BALB/c‐nude mice in the ratio of 1:1 to obtain F2 generation. The PCR primers were h‐ACE2‐MA‐sl 5′‐GGACAAGTTTAACACGAAGCC‐3′ and h‐ACE2‐MAas1 5′‐CAGCTGAAGCTTACATGAGAT‐3′. hACE2^+/−^ and hACE2^+/+^ transgenic nude mice (nude‐hACE2 mice) in F3 generation, obtained by crossing male hACE2^+/−^ nude mice with female hACE2^+/−^ hairy mice in F2 generation, were used in this study (Figure S1).^10^ Nude‐hACE2 mice were kept in a 12‐h light–dark cycle with free access to food and water. All the animal experiments were approved by the Institutional Animal Care and Use Committee of ILAS and PUMC (ILAS GH22001).

### 2.3 Protection of CoronaVac in nude‐hACE2 mice infected with WH‐09

Nude‐hACE2 mice (aged 3–4 months, nude‐hACE2/WV group, *n =* 12) were administered two doses (each 100 mL, on days 0 and 28) of CoronaVac via intramuscular injection in the hind legs. The control group (nude‐hACE2/W group, *n =* 12) was given an equal amount of phosphate‐buffered saline (PBS). To estimate the protective effect of CoronaVac vaccination on nude‐hACE2 mice against SARS‐CoV‐2 (WH‐09) infection, 100 TCID_50_ of WH‐09 diluted in 50 μL of PBS was intranasally inoculated under anesthesia using tribromoethanol (200 mg/kg) after the last dose of vaccination. After infection, six mice from each group were randomly selected for monitoring body weight changes and disease signs daily, including ruffled fur, hunched breathing, lethargy, and labored breathing. Each symptom was scored as one point. An additional six mice were euthanized for tissue sample collection and histopathological analysis at the time of imminent death (3–4 dpi [days postinfection]).

### 2.4 Protection of CoronaVac in nude‐hACE2 mice infected with the Omicron variant

Considering the mutation and weakening of the virulence of SARS‐CoV‐2 during the COVID‐19 transmission, we evaluated the protective effect of CoronaVac against Omicron variant infection in nude‐hACE2 mice. Twelve 3‐ to 4‐month‐old nude‐hACE2 mice (nude‐hACE2/OV) were administered three doses (each 0.1 mL, on days 0, 28, and 56) of CoronaVac via intramuscular injection in the hind legs. Twelve controls (nude‐hACE2/O group) were mock injected with the same volume of PBS. Two groups of nude‐hACE2 mice were anesthetized with tribromoethanol (200 mg/kg) and then intranasally infected with 100 TCID_50_ SARS‐CoV‐2 on day 14 after the last immunization. Six infected mice in each group were monitored for body weight and survival rate. General clinical symptoms, such as ruffled fur, hunched postures, labored breathing, and decreased response to external stimuli, were observed, and one point was scored for each symptom. The other six mice in each group were euthanized at the time of dying postinfection (or 4 dpi) to collect tissues for histopathological analysis.

### 2.5 Determination of viral gene copies in mouse tissues

Total RNA was extracted from homogenized lung samples. Tissue homogenates (1 g/mL) were prepared by homogenizing the perfused tissues using an electric homogenizer for 2 min 30 s in Dulbecco's modified Eagle's medium (DMEM). The homogenates were then centrifuged at 1509 *g* for 10 min at 4°C; the supernatant was collected and stored at −80°C for viral load detection. Quantitative real‐time PCR (qRT‐PCR) was performed using gene‐specific primers to determine the copy number of the SARS‐CoV‐2 RNA‐dependent RNA polymerase (*RdRp*) gene. To determine the viral load, total RNA was extracted from 350 µl of clarified tissue homogenates using an RNA extraction kit. qRT‐PCR was performed on a LightCycler 96 system using a one‐step RT‐PCR reaction kit with SARS‐CoV‐2 RdRp primers.

### 2.6 Live virus neutralization assays

Cytopathic effect (CPE) was used to detect neutralizing antibody (NAb) titers in serum. Serum samples were heat inactivated at 56°C for 30 min and diluted twofold using cell culture medium. Heat‐inactivated serum was mixed with a viral suspension containing 100 TCID_50_ in the ratio of 1:1 in 96‐well plates and incubated for 1 h at 36.5°C in a 5% CO_2_ incubator. Then, 1 × 10^4^ Vero E6 cells were added to the serum–virus mixture and incubated for 72 h after washing away the inoculum with PBS. The CPE was observed, and the NAb titer was calculated using the dilution number of 50% protective condition.

### 2.7 Histopathology and immunohistochemistry studies of mouse tissue sections

Formalin‐fixed and paraffin‐embedded tissues were cut into 4‐μm slices and stained with hematoxylin and eosin (H&E) for histopathological examination. The expression of the virus antigen in tissue was detected using mouse anti‐SARS‐CoV‐2 S protein antibodies, followed by staining with goat anti‐mouse IgG secondary antibody. The expression of hACE2 antigen in tissues was detected using staining with an rabbit anti‐hACE2 antibody, followed by staining with goat anti‐rabbit IgG secondary antibody.

### 2.8 Statistical analysis

All data were analyzed using GraphPad Prism 8.0 software. Two‐tailed unpaired Student's *t*‐test was used to compare the differences between the two groups, whereas one‐way analysis of variance (ANOVA) was used to compare data among the three groups. Statistical significant difference was defined as *p < 0.05 and **p < 0.01.

## 3 RESULTS

### 3.1 CoronaVac did not improve clinical symptoms in nude‐hACE2 mice infected with WH‐09

**FIGURE 1:**
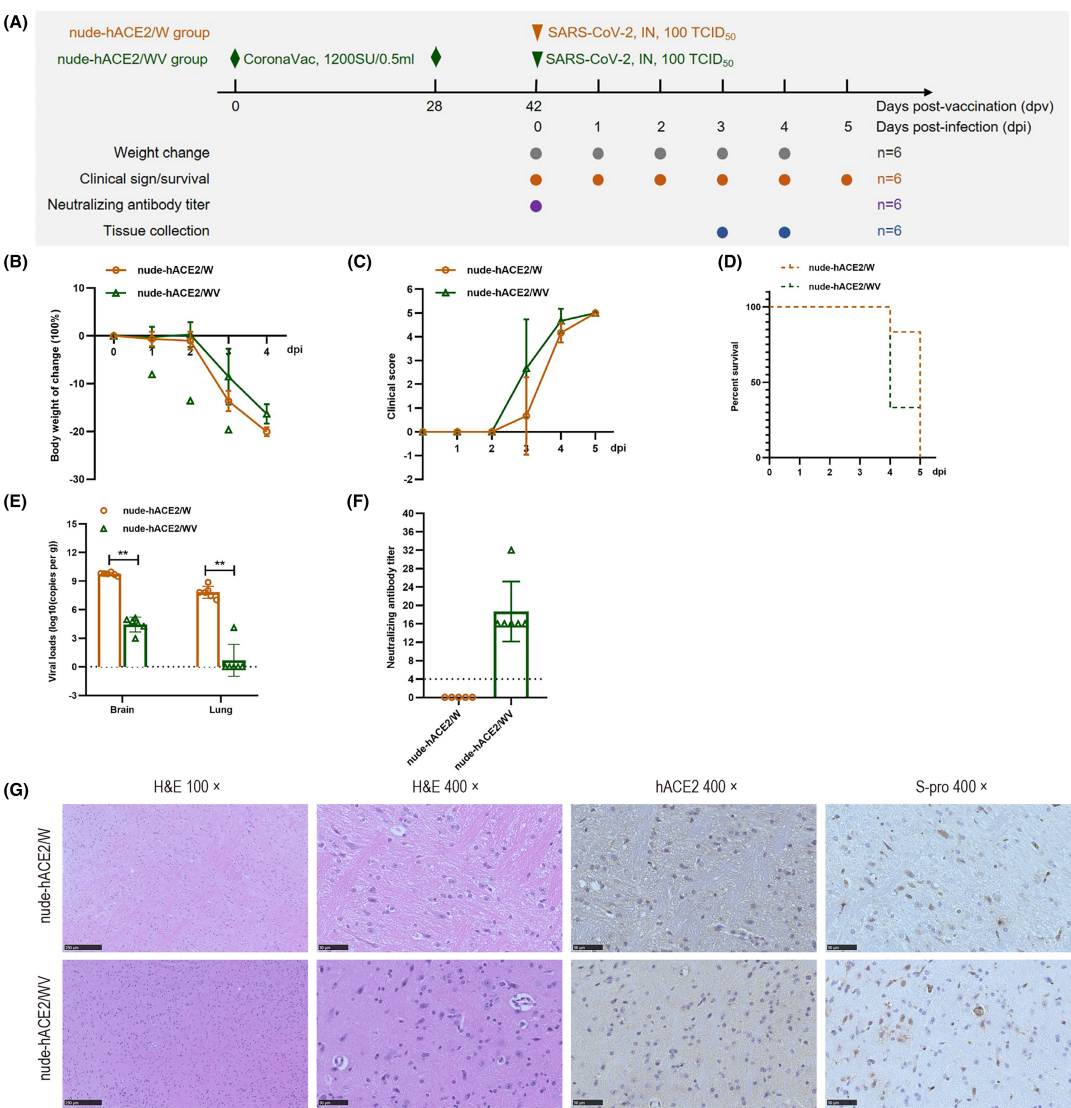
Clinical characteristics, virus replication, antibody detection, and brain histopathological characteristics in nude‐hACE2 mice infected with WH‐09 after vaccination. (A) Experimental design and sample collection. Twelve nude‐hACE2 mice (nude‐hACE2/WV group) were inoculated with 0.1 mL of CoronaVac two times (on days 0 and 28) and challenged intranasally with WH‐09 at 100 TCID_50_ at 14 days after the last dose of vaccination. The control group (nude‐hACE2/W group, *n* = 12) was infected intranasally with the same dose of WH‐09. Six infected animals were selected to record body weight and percent survival post‐infection. Tissue samples were harvested for virological and histopathology analyses in the other six dying mice. (B) Nude‐hACE2/W and nude‐hACE2/WV mice were observed for changes in body weight at the indicated time points. (C) General clinical symptoms were observed and scored by giving one score to each sign at the indicated time points. (D) Percentage survival at the indicated time points. (E) The viral RNA loads of dying nude‐hACE2 mice were quantified using RT‐PCR (3–4 dpi) (**p < 0.01). (F) The NAb (neutralizing antibody) titers of nude‐hACE2 mice at 0 dpi were also tested. (G) Histopathological characteristics and immunohistochemistry of the brain in nude‐hACE2 mice at 4 dpi were analyzed. Horizontal dashed lines indicate the detection limit of the viral RNA loads and NAb titers.

To evaluate the protective effect of CoronaVac on nude‐hACE2 mice infected with WH‐09, 24 mice were used in this study. The experimental design and longitudinal sampling plan are shown in Figure 1A. Twelve nude‐hACE2 mice (nude‐hACE2/WV group) were inoculated with 0.1 mL of CoronaVac two times (on days 0 and 28) and intranasally infected with WH‐09 at 100 TCID_50_ at 14 days after the final immunization. The control group (nude‐hACE2/W group, *n =* 12) was infected intranasally with the same dose of WH‐09. Nude‐hACE2/W mice showed obvious clinical symptoms and lost 13.6% body weight at 3 dpi. The mice lost a maximum of 20.08% body weight at 4 dpi (Figure 1B) and exhibited hunched posture, dyspnea, slow movement, and decreased response to external stimuli (Figure 1C), which all died at 5 dpi (Figure 1D). Compared with nude‐hACE2/W mice, nude‐hACE2/WV mice lost 8.55% and 16.35% body weight at 3 and 4 dpi (Figure 1B), accompanied by obvious clinical features, including ruffled fur, hunched posture, labored breathing, and decreased response to external stimuli (Figure 1C). All mice died on 5 dpi (Figure 1D).

To further evaluate the virus replication and histopathological changes in nude‐hACE2 mice, we harvested samples from the infected mice at 3–4 dpi for PCR, H&E staining, and immunohistochemistry staining. The mean viral RNA loads in the lungs and brain of nude‐hACE2/W mice were 10^7.83^ and 10^9.75^ copies/g (Figure 1E). The copy number of SARS‐CoV‐2 S protein in the brain of nude‐hACE2/W mice was significantly higher than that in the lungs (*p* < 0.01; Figure 1E), which was consistent with the results of nude‐hACE2 mice.^11^ Compared with nude‐hACE2/W mice, the viral load in the brain tissue of nude‐hACE2/WV mice significantly decreased (*p* < 0.01) but not eliminated. Significantly, the viral load in the lung tissue of five of six nude‐hACE2/WV mice was reduced to an undetectable level (Figure 1E). The NAb titers of the nude‐hACE2/WV mice were 16–32 arbitrary unit (AU) at 0 dpi (Figure 1F), which was 16.5‐fold lower than that in hACE2.^6^

The WH‐09 can infect the central nervous system of hACE2 mice,^12^ in which we can detect a high level of viral load. The brain tissue was collected for H&E staining and immunohistochemical study to further study the pathological changes and virus infection in nude‐hACE2 mice infected with WH‐09. Consistent with virus load results, the immunohistochemical staining results of hACE2 and SARS‐CoV‐2 S protein showed that the brain tissues of nude‐hACE2 mice expressed abundant hACE2 protein, and SARS‐CoV‐2 S protein was significantly distributed in the brain tissues in both groups (Figure 1G).

### 3.2 CoronaVac slightly improved the histopathological characteristics in nude‐hACE2 mice infected with WH‐09

**FIGURE 2:**
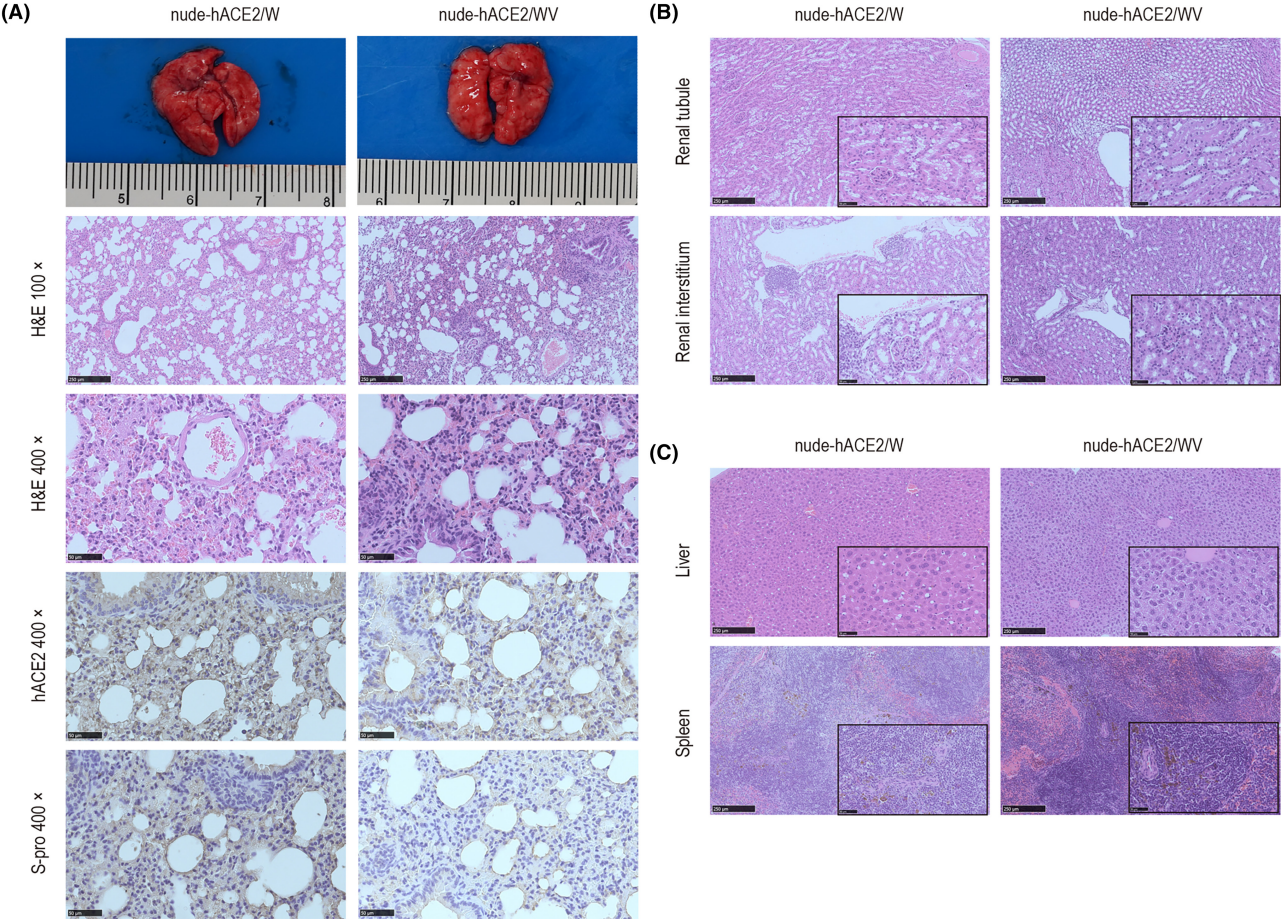
Histopathological characteristics of the lung, renal interstitium, renal tubules, liver, and spleen in nude‐hACE2/W and nude‐hACE2/WV groups infected with WH‐09. The tissue samples of nude‐hACE2/W and nude‐hACE2/WV mice were collected, fixed in formalin, and embedded in paraffin for histopathological examination. (A) Histopathological characteristics and immunohistochemistry of lung in nude‐hACE2 mice at 4 dpi were analyzed. Histopathological characteristics of (B) renal tubules and renal interstitium and (C) liver and spleen.

The main complications caused by COVID‐19 are pneumonia and acute respiratory distress syndrome, which may also cause other complications, including acute liver and kidney injury, as well as secondary infection and inflammatory reaction. The histopathology of the lung, kidney, liver, and spleen was evaluated. The lungs of nude‐hACE2/W mice showed moderate interstitial inflammation as thickening of alveolar septa, peribronchiolar and perivascular inflammatory infiltration, and mild collagen fiber hyperplasia (Figure 2A). The immunohistochemistry results of lungs showed that hACE2 was highly expressed in epithelial cells and bronchioles in lungs, and S protein was abundantly expressed in bronchiolar epithelial cells, alveolar epithelial cells, and alveolar macrophages (Figure 2A), consistent with H&E findings. The lungs of nude‐hACE2/WV mice demonstrated a mild degree of alveolar wall infiltration and blood vessel congestion, and SARS‐CoV‐2 S protein was also reduced in bronchiolar epithelial cells, alveolar epithelial cells, and alveolar macrophages, which were comparable or milder compared to those observed in nude‐hACE2/W mice (Figure 2A).

The histopathological results in other organs of nude‐hACE2/W mice showed vacuolar degeneration in renal tubular cells, inflammatory cell infiltration in the renal interstitium and perivascular (Figure 2B), swelling of Ito cells in liver tissue, and more apoptotic lymphocyte cells in the splenic germinal center (Figure 2C). Compared with nude‐hACE2/W mice, there were no obvious pathological changes in the renal tubules, renal interstitium, and spleen of nude‐hACE2/WV mice. Besides, liver tissue of nude‐hACE2/WV mice showed mild swelling and local vacuolar degeneration.

### 3.3 CoronaVac had no significant improvement in clinical symptoms in nude‐hACE2 mice infected with the Omicron variant

**FIGURE 3:**
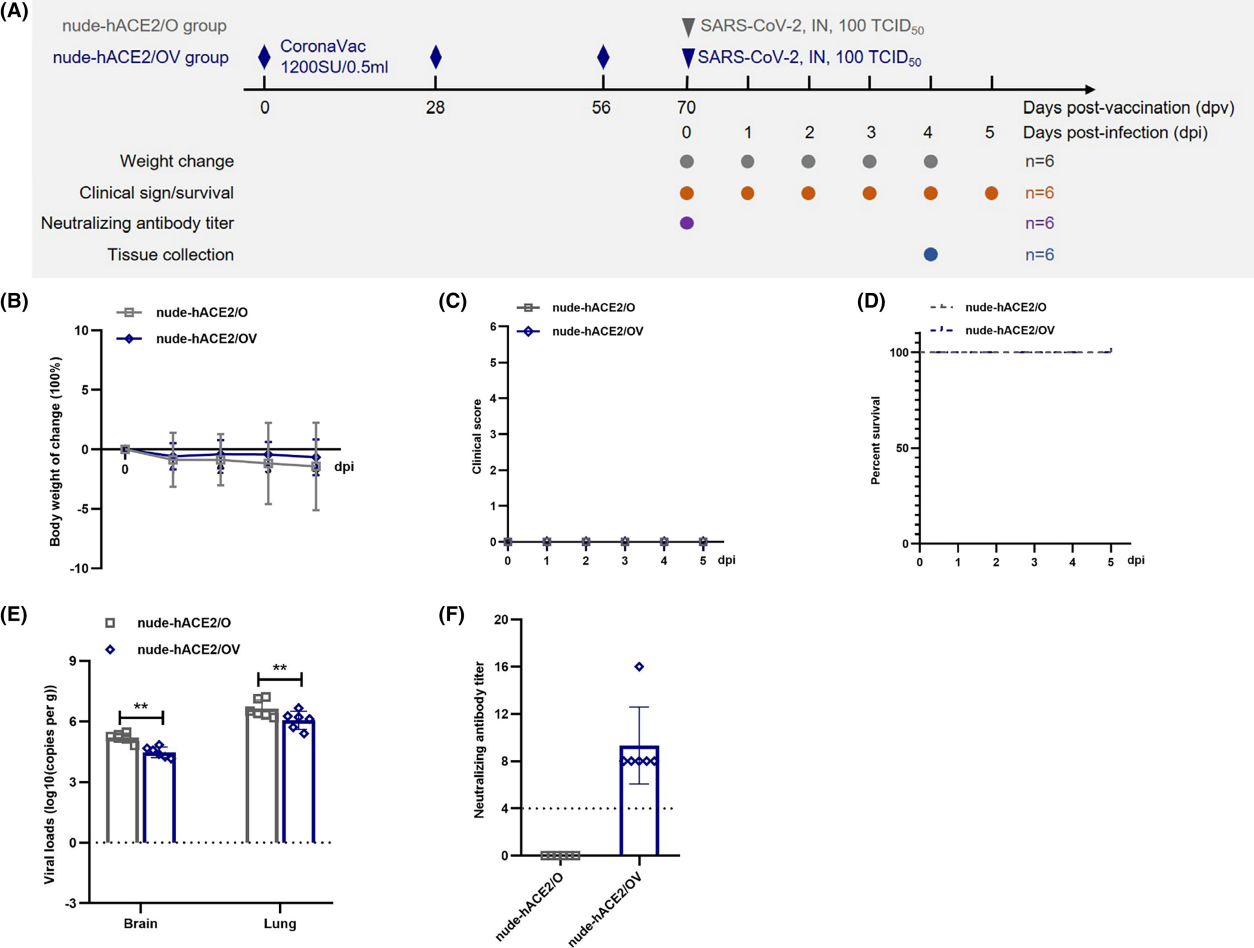
The protective ability of CoronaVac on nude‐hACE2 mice infected with the Omicron variant. (A) Experimental design and sample collection. Twelve 3‐4‐month‐old nude‐hACE2 mice (nude‐hACE2/OV group) were injected intramuscularly into the hind legs with three doses (on day 0, 28, and 56) of 0.1 mL CoronaVac. As controls, nude‐hACE2/O mice (*n* = 12) were mock‐immunized with the same volume of PBS. 100 TCID_50_ Omicron variant diluted in 50 μL of PBS were intranasally inoculated in both two groups under anesthesisa by tribromothanol (200 mg/kg). (B) Body weight change at the indicated time points. (C) Disease signs, including ruffled fur, hunched posture, labored breathing, and decreased response to external stimuli, were observed and scored by giving one score to each sign at the indicated time points. (D) Percentage survival at the indicated time points. (E) The viral RNA loads of nude‐hACE2 mice were quantified using RT‐PCR of dying mice (or 4 dpi) (**p < 0.01). (F) The NAb (neutralizing antibody) titers of nude‐hACE2 mice at 0 dpi were also tested. Horizontal dashed lines indicate the detection limit of the viral RNA loads and NAb titers.

Considering the pathogenicity of the SARS‐CoV‐2 variant weakened during the COVID‐19 transmission^13^ and the Omicron variant vaccine unpublished, the currently approved vaccine is still a strategy to reduce the serious disease and high mortality caused by the current epidemic virus strain. The NAb titers in the serum of nude‐hACE2 mice immunized with two doses of CoronaVac were only 16–32 AU, which is far lower than that in normal hACE2 mice, and showed limited protection against WH‐09. Nude‐hACE2 mice (nude‐hACE2/OV group) were injected thrice (on days 0, 28, and 56) with 0.1 mL of CoronaVac via intramuscular injection in the hind legs to increase the antibody titer in mice. Nude‐hACE2/O and nude‐hACE2/OV groups were anesthetized with tribromoethanol (200 mg/kg) and then intranasally infected with 100 TCID_50_ Omicron variants on day 14 postimmunization. Nude‐hACE2/O mice showed normal body weight changes and clinical symptoms at 1–5 dpi (Figure 3B,C), and the survival rate was 100% at 5 dpi (Figure 3D). Compared with the nude‐hACE2/O, the clinical characteristics of nude‐hACE2/OV mice were normal.

To further evaluate the virus replication of nude‐hACE2/O and nude‐hACE2/OV, we harvested samples for PCR at 4 dpi. The mean viral RNA loads were 10^6.63^ and 10^5.2^ copies/g in the lungs and brain of nude‐hACE2/O mice and were 10^6.06^ and 10^4.48^ copies/g in the lungs and brain of nude‐hACE2/OV mice. Compared with nude‐hACE2/O, the viral load in the lung tissue of nude‐hACE2/OV mice was significantly reduced (*p* < 0.01) but not eliminated (Figure 3E). The NAb titers of the nude‐hACE2/OV mice were 8–16 AU at 0 dpi (Figure 3F).

### 3.4 CoronaVac did not improve the histopathological characteristics in nude‐hACE2 mice infected with the Omicron variant

**FIGURE 4:**
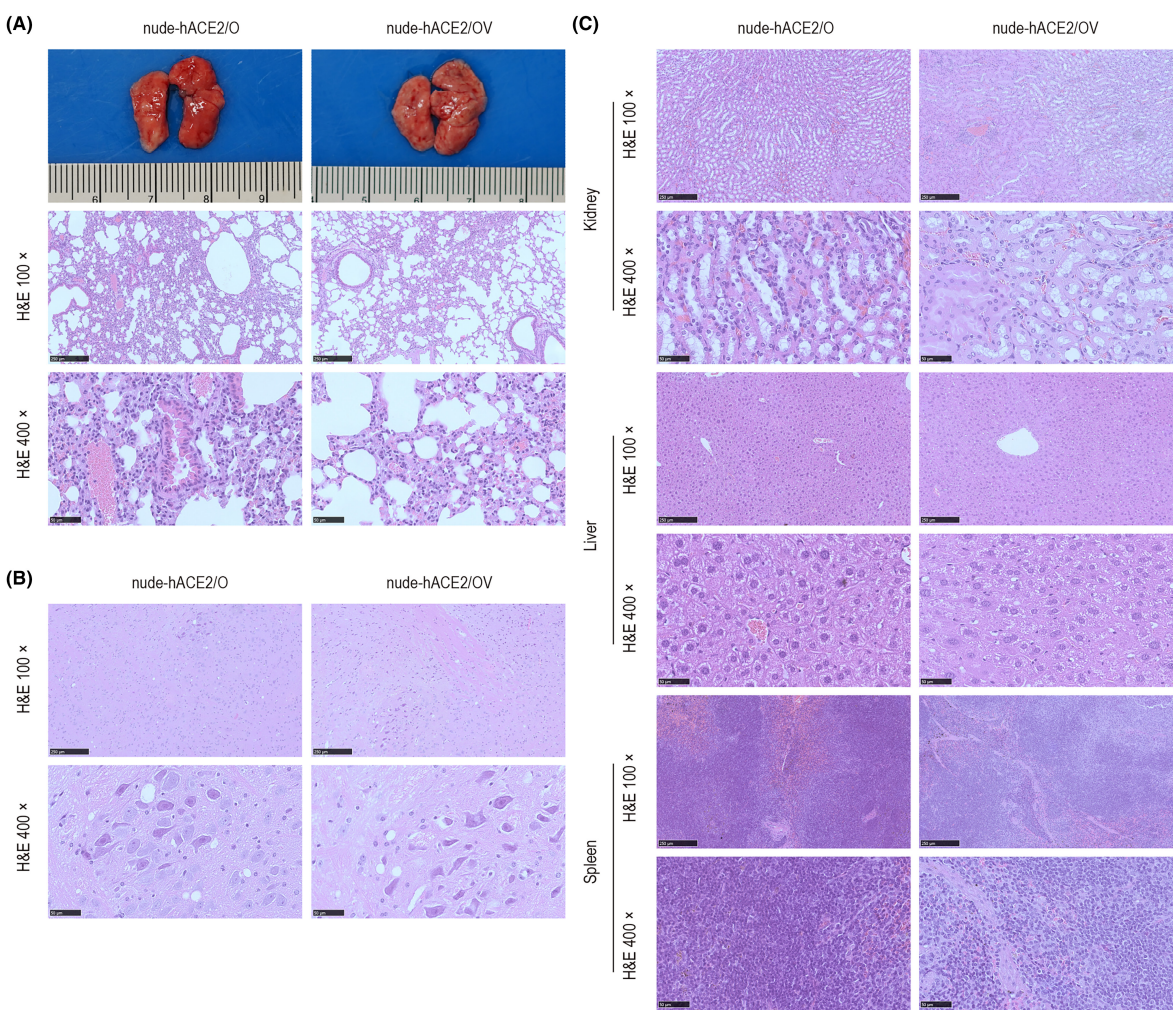
Histopathological characteristics of brain, lungs, renal interstitium, renal tubules, liver, and spleen in nude‐hACE2/O and nude‐hACE2/OV groups infected with the Omicron variant. The tissue samples of mice were collected, fixed in formalin, and embedded in paraffin for histopathological examination. (A) Histopathological characteristics and immunohistochemistry of lungs in nude‐hACE2 mice at 4 dpi were analyzed. Histopathological characteristics of (B) brain and (C) kidney, liver, and spleen.

To further study the histopathological changes in nude‐hACE2 mice infected or postimmune infected with the Omicron variant, we collected the brain and lung tissues for H&E staining. The lungs of nude‐hACE2/O mice showed mild interstitial pneumonia, including mild and focal thickening of alveolar septa, peribronchiolar and perivascular mild inflammatory infiltration, and mild collagen fiber hyperplasia (Figure 4A). The brain tissue of nude‐hACE2/O mice showed local vacuolar degeneration, neuronal degeneration and necrosis, and dissolved nucleus. Nude‐hACE2/OV mice showed the same histopathological features in the brain and lungs as nude‐hACE2/OV mice (Figure 4B). CoronaVac did not improve the histopathological changes in nude‐hACE2/O and nude‐hACE2/OV mice. There were no obvious pathological changes in the kidney, liver, or spleen of both groups (Figure 4C).

## 4 DISCUSSION

Immunocompromised individuals often experience inflammation and are at an increased risk of contracting SARS‐CoV‐2. They are more likely to develop severe illness as a result of infection.^8^, ^14^, ^15^, ^16^ Rodents have been widely used to study the pathogenesis and vaccination response of SARS‐CoV‐2 due to their wide availability and short growth cycle. Athymic nude mice are a murine strain with a spontaneous deficiency of the *Foxn1* gene, which can result in thymic deterioration or its absence, leading to immunosuppression and a decrease in the number of T cells. This model is widely used in preclinical evaluations of immunocompromised populations and in the study of infectious diseases. However, the WH‐09 of SARS‐CoV‐2 that was prevalent in the early stages of COVID‐19 had a low binding efficiency with mACE2, and infected athymic nude mice showed almost no symptoms of the disease.^17^

In this study, we used 3‐ to 4‐month‐old nude‐hACE2 mice as research objects to investigate the protection of CoronaVac against WH‐09 infection. First, we demonstrated that nude‐hACE2 mice infected with WH‐09 showed obvious clinical symptoms such as weight loss, hunched back, movement retardation, and respiratory distress. The replication of the virus in lung and brain tissues was obvious and showed corresponding histopathological changes, which was consistent with the hACE2 mice model. Second, CoronaVac reduced viral load replication and alleviated the histopathological changes in nude‐hACE2/WV mice. Compared with nude‐hACE2/W, the viral load was significantly reduced in brain tissue and was reduced to an undetectable level in the lungs of nude‐hACE2/WV mice. The histopathological changes in the brain, lungs, kidneys, and liver in nude‐hACE2/WV mice subsided. Third, the NAb titers in the serum of nude‐hACE2 mice with two doses of CoronaVac were significantly lower than those in wide‐type hACE2 mice. Overall, our research showed that CoronaVac inoculation can partly alleviate the symptoms in nude‐hACE2 mice infected with WH‐09, suggesting that CoronaVac can play a certain role in the prevention of critically ill patients.

During COVID‐19 transmission, emerging SARS‐CoV‐2 variants, including the Delta and Omicron variants, reduced their fusogenicity and pathogenicity.^18^ The titers of NAbs in the serum of nude‐hACE2 mice immunized with two doses of CoronaVac were far lower than those in normal hACE2 mice and showed limited protection against WH‐09. Therefore, we further studied the protective effect of booster vaccination on nude‐hACE2 mice infected with the Omicron variant. First, compared with the WH‐09 infection, nude‐hACE2 mice infected with the Omicron variant showed no obvious clinical symptoms and lethality, which showed only mild histopathological changes in lung and brain tissues, including mild interstitial pneumonia and local vacuoles in brain tissue. However, the replication of the virus in lung and brain tissues was obvious. Second, compared with nude‐hACE2/O mice, the viral load in the lung and brain tissue of nude‐hACE2/O mice decreased, and mild histopathological features were still observed in the lungs and brain, indicating that vaccination reduced virus replication in mice. Third, the NAb titers of the Omicron variant of nude‐hACE2/OV mice with three doses of vaccination were lower than those of nude‐hACE2/WV mice. Lv et al. found that in inactivated vaccine sera, at 1–3 months post three doses, geometric mean titers of NAb against the Omicron variant were 4.9/5.2‐fold lower than those of the WH‐09, which was consistent with the results of this study.^19^ The infection symptoms of the Omicron variant were mild, and no obvious histopathological changes in nude‐hACE2 mice were observed after vaccination.

In general, our research shows that CoronaVac conferred some protection among the nude‐hACE2 mice infected with the WH‐09 or Omicron variant. However, in this experiment, we chose the antibody peak time to carry out the study, as the time point of infection postimmunization in the population is uncertain. Therefore, it is still necessary to further carry out long‐term follow‐up research in animals.

## AUTHOR CONTRIBUTIONS

**Hong Gao**: Conceptualization, resources, methodology, and supervision: **All authors**: Investigation. **Kaili Lin**: Writing of the original draft. **Kaili Lin** and **Hong Gao**: Review and editing. **Hong Gao**: Funding acquisition.

## FUNDING INFORMATION

This project was supported by the National Key Research and Development Project of China (grant number: 2020YFA0707600).

## CONFLICT OF INTEREST STATEMENT

The authors declare no competing financial interests or other associations that may pose a conflict of interest (e.g., pharmaceutical stock ownership, consultancy).

## ETHICS STATEMENT

All the animal experiments were approved by the Institutional Animal Care and Use Committee of ILAS & PUMC (ILAS GH22001).

## Supporting information


Figure S1
