# Supplementary figures and images for: The protection of CoronaVac against the infection of wild‐type SARS‐CoV‐2 (WH‐09) or Omicron variant in nude‐hACE2 mice
Source: Animal Model Exp Med. 2023 Jul 10;6(4):346–54. doi: 10.1002/ame2.12336 (PMC10486324; doi:10.1002/ame2.12336)

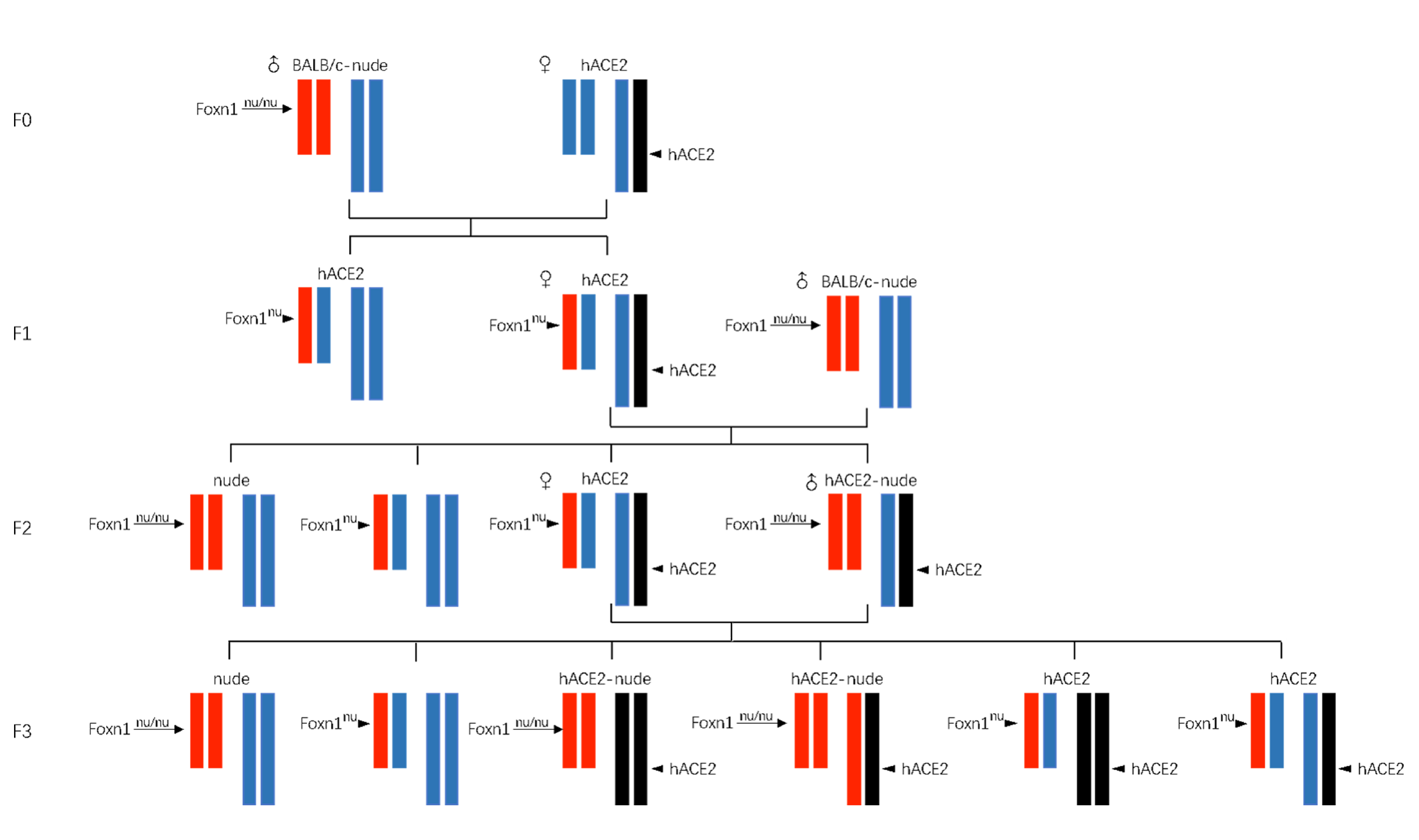

Supplement: Supplementary file 1 — Figure S1 [file AME2-6-346-s001.tif]
